# Trends in surgical indications and causative diagnoses in enucleation from 2007 to 2022

**DOI:** 10.1038/s41598-025-13975-4

**Published:** 2025-08-01

**Authors:** Nicolas Pensel, Siegfried Priglinger, Christoph Hintschich, Anna Schuh

**Affiliations:** Department of Ophthalmology, Ludwig-Maximilians-University Munich, Mathildenstr. 8, 80336 Munich, Germany

**Keywords:** Enucleation, Evisceration, Painful blind eye, Trauma, Surgery, Medical research

## Abstract

To evaluate surgical indications and causative underlying diseases in patients undergoing enucleation in a tertiary eye unit. Retrospective analysis of all enucleations performed at the University Eye Hospital of LMU Munich from January 2007 to December 2022. 491 eyes of 491 patients were enucleated in this period; 237 right and 254 left eyes. 59.3% (291) of patients were male, while 40.7% (200) were female. The median patient age at enucleation was 59 years (range 2–99, IQR 43–72). The four most common surgical indications were painful blind eye (318, 64.8%), malignancy (139, 28.3%), disfiguring blind eye (14, 2.9%) and treatment-refractory perforated corneal ulcer (13, 2.6%). There was only one indication for enucleation due to acute trauma (1, 0.2%). Information on causative diagnosis was available from 2013 to 2022 (257). The most common causative diagnoses leading to enucleation were choroidal melanoma (107, 41.6%), status post (s/p) trauma (65, 25.3%), and s/p retinal detachment (17, 6.6%). The annual enucleation count showed a decline from 2007 to 2022. Regarding indications for enucleation there is a negative trend for “painful blind eye” and “malignant tumor”. Our study demonstrates a decrease in the annual number of enucleations between 2007 and 2022. While the causative diagnoses remained unchanged over the last ten years, there was a negative trend in surgical indications due to malignant tumors and painful blind eyes. Only one enucleation was performed due to acute trauma.

## Introduction

Enucleation remains a definitive therapeutic option for a range of severe ocular conditions, including intraocular malignancies, severe eye trauma or end-stage ocular diseases unresponsive to other treatments^1^. The age- and sex-adjusted incidence of enucleation in Western populations has been reported to range between 1.48 and 4.32 per 100,000 residents^2,3^. From the late twentieth century through 2013, retrospective studies assessed the frequency, indications and underlying diagnosis associated with enucleations of the globe, primarily in population-based or tertiary care settings^2–6^.

While some studies observed a decrease in annual enucleation rates, attributed to advances in diagnostic and therapeutic modalities, findings have not been entirely consistent across countries and healthcare systems. For example, Geirsdottir et al.^3^ reported a downward trend for eye amputations in Iceland, as did Erie et al.^4^ for Minnesota. In contrast Rasmussen et al.^5^ noted overall stable numbers with a relative increase in eviscerations in a Danish tertiary center. Across all studies, the most frequent common surgical indications in these earlier studies included painful blind eyes, suspected intraocular malignancy, acute trauma and infection^2–6^.

The most recent study on this topic, an institutional analysis from Western Canada including data up to 2016, reported stable numbers of enucleations^7^. In contrast, European data extended only up to 2004^3^, leaving current trends insufficiently defined. In parallel, significant therapeutic advances, including anti-VEGF therapy^8^, improved medical and therapeutical management and more effective trauma care and prevention^9^, have transformed ophthalmic disease management over the past decades.

The present study aims to provide an updated, comprehensive analysis of enucleations performed at a European tertiary eye center over a 16-year period, with a focus on trends in annual procedure numbers, surgical indications and underlying causative diagnoses.

## Methods

We conducted a retrospective review of all patients who underwent enucleation at the University Eye Hospital of LMU Munich over a 16-year period from January 1, 2007, to December 31, 2022. Patient data were obtained through systematic analysis of medical records, historical surgical logs and the institutional pathology database. The collected variables included patient age, sex, date of enucleation, side of the enucleated eye, surgical indication for enucleation and histopathological diagnosis.

For the 10-year period from January 1, 2013, to December 31, 2022, detailed clinical information was available, including the underlying condition leading to enucleation (causative diagnosis) and best-corrected visual acuity at the time of surgical indication.

For cases enucleated between January 1, 2007, and December 31, 2012, access to patient records was limited due to incomplete data migration during the digitization of hospital archives. Consequently, information on the underlying diagnosis and visual acuity at the time of indication was not consistently available and was therefore excluded from the analysis for this subgroup.

Cases of evisceration and exenteration were excluded from the study throughout the entire study period.

This study was planned as a retrospective analysis and received approval from the Institutional Review Board of Ludwig-Maximilians-University Munich (reference number 23-0540). Due to the retrospective nature of the analysis, the Institutional Review Board of Ludwig-Maximilians-University Munich waived the requirement for informed patient consent. We adhered to the principles of the Declaration of Helsinki throughout the study.

### Statistical analysis

Data was collected in MS-Excel 2022 spread sheets (Microsoft Corporation, Unterschleissheim, Germany). The data was analyzed by Wilcoxon rank-sum-test, t-test and Mann–Kendall test with sieve-bootstrap enhancement to test for possible trends. The Mann–Kendall test with sieve-bootstrap enhancement was applied to assess monotonic trends and a sieve-bootstrap t-test to evaluate linear trends in annual enucleation counts, accounting for potential temporal autocorrelation. These methods provide robust p-values for detecting linear and non-linear trends in small time series. P-values were derived using a sieve-bootstrap procedure, which generates an empirical distribution of the test statistic by resampling the residuals of an autoregressive model, thereby preserving the time dependence structure of the data. A p-value < 0.05 was considered statistically significant.

## Results

### Demographic data and visual acuity

A total of 491 eyes from 491 patients underwent enucleation between 2007 and 2022, including 237 right eyes and 254 left eyes. The median annual number of enucleations was 29.5 (IQR 24–36). Of these patients, 59.3% (291) were male and 40.7% (200) were female. The median age of patients at the time of enucleation was 59 years (range 2–99, IQR 43–72).

Best corrected visual acuity (BCVA) of 257 patients revealed that 64.2% (165) had no light perception, light perception in 22.9% (59), hand motion 8.6% (22), counting fingers 0.4% (1), < 0.05 1.6% (4), > 0.05 2.3% (6).

### Surgical indications

The four most common surgical indications were painful blind eye (318, 64.8%), malignancy (139, 28.3%), disfiguring blind eye (14, 2.9%), and treatment-refractory perforated corneal ulcer (13, 2.6%). Only one enucleation was performed due to acute trauma (1, 0.2%). (Table 1).

**Table 1:** List of all surgical indications for enucleation from 2007 to 2022.

Surgical indication (2007–2022)	Number (total 491)	%
Painful blind eye	318	64.8
Malignancy	139	28.3
Disfiguring blind eye	14	2.9
Corneal perforation	13	2.6
Endophthalmitis	4	0.8
Risk for sympathetic ophthalmia	2	0.4
Acute trauma	1	0.2

### Causative diagnosis

**Fig. 1:**
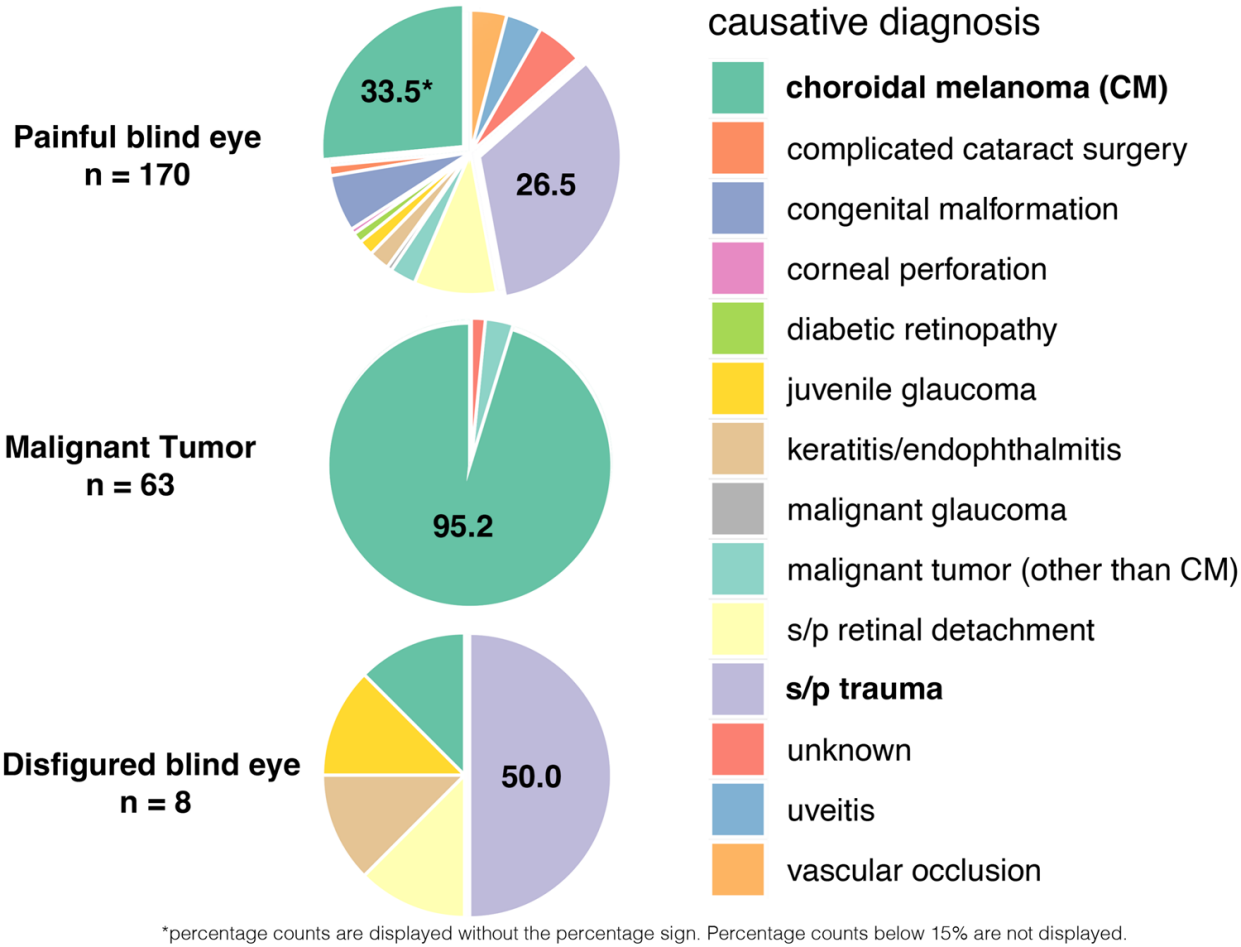
Distribution of causative diagnoses by surgical indication. The three main surgical indications categorized by causative diagnoses for enucleation in all patients who underwent enucleation during 2013–2022, displayed in pie charts.

For the subgroup of 257 patients enucleated between 2013 and 2022 detailed information on causative diagnoses were available. The most frequent causative diagnoses leading to enucleation were choroidal melanoma (107, 41.6%), status post (s/p) trauma (65, 25.3%), and s/p retinal detachment (17, 6.6%). (Table 2). The distribution of causative diagnoses by surgical indications is illustrated in (Fig. 1).

**Table 2:** List of all causative diagnoses for enucleation from 2013 to 2022.

Causative diagnosis (2013–2022)	Number (total 257)	%
Choroidal melanoma	107	41.6
s/p trauma	65	25.3
s/p retinal detachment	17	6.6
Congenital malformation	11	4,3
Vascular occlusion	10	3.9
Inflammation (severe keratitis, endophthalmitis)	8	3,1
Uveitis	7	2.7
Malignancy (other than choroidal melanoma)	7	2.7
Complicated cataract surgery	4	1.6
Juvenile glaucoma	4	1.6
Diabetic retinopathy	2	0.8
Perforated corneal ulcera	1	0.4
Malignant glaucoma	1	0.4
Unknown	14	5.4

### Trends from 2007 to 2022

**Fig. 2:**
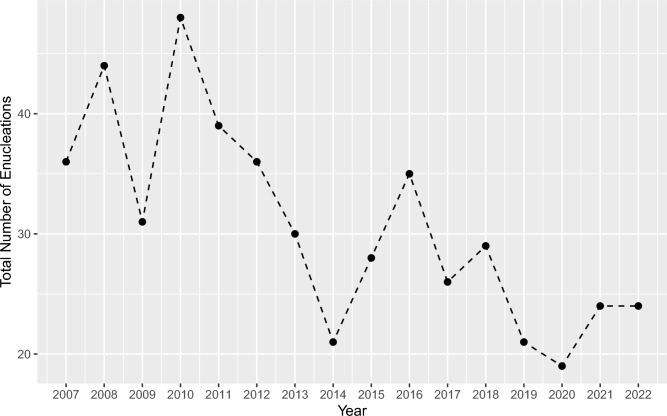
Annual number of enucleations from 2007 to 2022. The x-axis represents the calendar year, the y-axis shows the total number of enucleations performed each year. The number of enucleations per year exhibited a consistent decrease from 2007 to 2022, with an overall percentage change of −33.3% (median annual change: −7.7%; IQR −22.2–23.6%, Student’s t =  −3.14, p < 0.001, sieve-bootstrap t-test).

The annual number of enucleations decreased over time with an overall reduction of -33.3% between 2007 and 2022. The median annual change was −7.7% (IQR -22.2% to 23.6%, Student’s t =  −3.14, p < 0.001, sieve-bootstrap t-test) (Fig. 2).

**Fig. 3:**
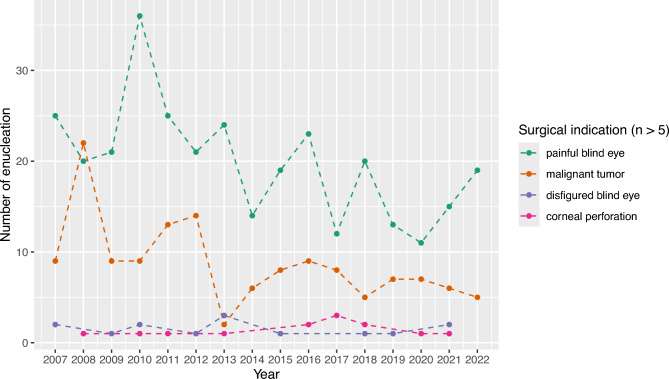
Annual number of enucleations by major surgical indication from 2007 to 2022. The x-axis represents the calendar year, the y-axis shows the total number of enucleations performed per year for each surgical indication. Each point indicates the annual count for an indication; dashed lines connect the points to illustrate trends over time. Surgical indications with fewer than five cases overall were excluded.

The annual median patient age remained stable throughout the study period at 58 years (IQR 55,5–60,5). Between 2007 and 2019 (excluding the Covid-19 pandemic years) enucleations for painful blind eyes (Z =  −0.458, p = 0.025, Mann–Kendall test with sieve-bootstrap enhancement) and malignancies (Z =  −0.417, p = 0.048) declined significantly, while other indications remained stable (Fig. 3).

The distribution of causative diagnoses did not change significantly over this period, as illustrated by the line diagram showing annual counts for each causative diagnosis in Supplementary Figure S1.

### Subgroup analysis

**Fig. 4:**
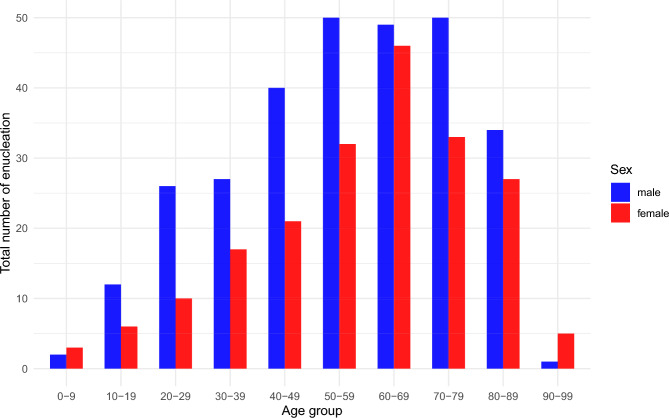
Distribution of total enucleations grouped by patient age and sex. Age groups were defined in 10-year increments (e.g., 0–9, 10–19, 20–29, etc.). The x-axis shows the age groups, the y-axis shows the total number of enucleations performed over the 16-year period (2007–2022). Data are further stratified by sex (blue = male and red = female).

Male patients predominated across nearly all age groups (Fig. 4). Age at surgery varied significantly by surgical indication: median age was 68 years (IQR 59.5–78) in patients with malignancy and 72 years (IQR 59–79) in those with corneal perforation, both significantly higher compared to painful blind eyes (median 53, IQR 36–69; p < 0.001 and p = 0.002, respectively, Wilcoxon rank-sum test) and disfiguring blind eyes (median 44, IQR 26.75–66; p = 0.002 and p = 0.013, respectively). These age distributions, stratified by sex, are illustrated in box plots shown in Supplementary Figure S2.

Age also differed significantly across causative diagnoses. Patients with choroidal melanoma had a median age of 69 years (IQR 58.5–77.5), which was higher than that of patients with prior trauma (median 44, IQR 32–55) and retinal detachment/surgery (median 50, IQR 40.25–60; both p < 0.001, Wilcoxon rank-sum test). These age distributions, stratified by sex, are illustrated in the box plots shown in Supplementary Figure S3. To investigate possible temporal trends, the dataset was divided into two five-year intervals (2013–2017 vs. 2018–2022) for subgroup analysis of causative diagnoses, including trauma and choroidal melanoma. Among patients with trauma, the median time from diagnosis to enucleation increased from 24 months (IQR 2–65) in 2013–2017 to 34 months (IQR 14–67, p = 0.3, Wilcoxon rank-sum test) in 2018–2022. For patients with choroidal melanoma, median times were 113 months (IQR 44–358) and 247 months (IQR 106–447, p = 0.11), respectively.

## Discussion

Over the 16-year study period, enucleation numbers declined, particularly for painful blind eyes (64.8%) and malignancies (28.3%). Acute trauma was a rare indication, with only one case recorded. Diabetic retinopathy (0.8%) and vascular occlusions (3.9%) accounted for a small proportion of underlying causative diagnoses. These trends support the hypothesis that advances in diagnostics, treatment and prevention have contributed to the overall decline in enucleations in the recent years.

Our study demonstrates a notable decline in the annual number of enucleations performed at our institution between 2007 and 2022, supporting the observations from previous studies over the past decades^2–4,6^. This trend likely reflects the increasing effectiveness of modern diagnostic and therapeutic options in preserving the globe and visual function.

Demographic findings in our study are largely in line with previous reports. We observed a male predominance (approximately 3:2) consistent with earlier studies where male patients accounted for 55.6 to 68% of enucleation cases^2–4,6^. The median age at enucleation in our cohort was 59 years, which is older than in earlier incidence-based studies (e.g. 46 years reported by Erie et al.^4^ and 51 years by Geirsdottir et al.^3^), but comparable to more recent center-based analyses (59.4 years in Chan et al.^6^). This may reflect general demographic shifts and increased life expectancy^10^. Our subgroup analysis revealed significant age differences across surgical indications and causative diagnoses. Patients undergoing enucleation due to malignant tumors or corneal perforations as surgical indication were significantly older than those with painful or disfiguring blind eyes.

Similarly, patients with malignant tumours were older than those with trauma- or retinal detachment-related underlying diagnoses. These patterns reflect the age distribution of the underlying conditions: ocular malignancies such as choroidal melanoma (median age at diagnosis 55 years reported by Singh^11^) and corneal perforation (mean age at diagnosis 61.1 years reported by Takahashi^12^) predominantly affect older adults, whereas painful or disfiguring blind eyes often result from trauma, repeated surgeries or early-onset conditions such as juvenile glaucoma or congenital diseases, which span a broader age range^13–17^. Furthermore, it may be of interest to investigate whether age at the time of enucleation influences long-term functional or psychosocial outcomes.

In line with prior literature^2–4,6^, the most frequent surgical indication for enucleation in our study was a painful blind eye (64.8%), followed by malignant tumors (28.3%). The slightly elevated rate of malignancy compared to prior population-based studies (e.g., 16^4^ and 20%^3^) likely reflects the referral bias of our center, which serves as a tertiary site for ocular oncology. Similarly, choroidal melanoma was the most common single causative diagnosis, accounting for 42% of all enucleations. This is higher than in population-based studies (17 to 24.5%^2–4^) but aligns with results from other tertiary centers (55.2^6^ and 69%^5^).

Posttraumatic conditions represented 25.3% of enucleations in our study, again comparable to other older center-based studies^5,6^, but notably lower than in previous incidence-based studies (39^3^ and 35%^4^).

Remarkably, only one single enucleation (0.2%) was performed for acute trauma, in stark contrast to historical data reporting 12–17.5%^2–4^. This may reflect improved primary surgical management, heightened awareness of protective measures in sports^18^, workplaces and daily life^19^ or a shift in clinical decision making aimed at globe preservation. In our department, surgical practice emphasizes reconstruction over primary enucleation in acute trauma whenever feasible, which may have influenced these results.

The decline in trauma-related enucleation in our recent study compared to older analysis marks a significant change. It highlights the potential impact of both preventive strategies and surgical advancements in acute care. Reduced enucleation rates for trauma may reflect broader public health improvements, including eye protection regulations and increased public awareness, although this cannot be directly confirmed by our data.

The majority of enucleation due to painful blind eye were secondary to trauma (33.5%) and choroidal melanoma (26.5%). Vascular occlusions and diabetic retinopathy represented only 4.2 and 1.2% of painful blind eyes, which is lower than in earlier reports (16.6^3^ to 22.6%^4^ and 3.3^3^ to 12.9%^4^, respectively). This supports the hypothesis that ophthalmological advancements, including anti-VEGF therapy^8^, improved surgical and early screening tools, have contributed to a reduced rate of end stage diseases necessitating enucleation.

While we observed a tendency toward longer intervals from diagnosis to enucleation in more recent years, this trend did not reach statistical significance. Nonetheless, the finding may as well reflect improvements in therapeutic options that enable longer preservation of the globe. Given the relatively short observation period and lack of earlier data in our cohort or comparable previous studies, no robust conclusions about temporal trends can be drawn at this stage. Future studies with longer follow-up will be needed to clarify whether advances in treatment have genuinely delayed the need for enucleation.

### Limitations

This study is subject to limitations inherent to its retrospective design. Trend analysis for causative diagnoses, were restricted by incomplete data from 2007 to 2013. The center-based nature and tertiary referral status of our department may introduce institutional and regional biases, limiting generalizability to the broader population. In our department enucleation is preferred over evisceration which limits comparability with previous studies including both procedures in their analyses. Furthermore, incidence data cannot be derived from our cohort.

## Conclusion

Our retrospective analysis confirms a decline in enucleation numbers over the past 16 years, consistent with earlier studies. The decrease was most evident for the indications of painful blind eyes and malignancies. Diabetic retinopathy and vascular occlusions were rare causative diagnoses. Only one case involved acute trauma as indication for enucleation. These trends likely reflect advances in diagnostics, treatment and prevention.

This study provides an up-to-date overview of current enucleation indications in a European tertiary care setting and underscores the impact of preventive and therapeutic advancements on clinical practice. Multicenter, population-based analyses are needed to validate these results and assess regional or institutional differences. Future research might also investigate clinical decision processes leading to enucleation versus eye-preserving interventions.

## Supplementary Information

Supplementary Information.

## Data Availability

The data that support the findings of this study are available from the corresponding author, [A.S.], upon reasonable request.
