# Supplementary material for: Trends in surgical indications and causative diagnoses in enucleation from 2007 to 2022
Source: Sci Rep. 2025 Aug 1;15:28120. doi: 10.1038/s41598-025-13975-4 (PMC12317067; doi:10.1038/s41598-025-13975-4)

Article title: Surgical indications and causative diagnoses in enucleation - a 16-year analysis.

Journal name: Scientific Reports

Authors and Affiliation:

Nicolas Pensel, MD[1]

Siegfried Priglinger, MD[1]

Christoph Hintschich, MD[1]

Anna Schuh, MD[1]

[1] Ludwig-Maximilians-University Munich, Department of Ophthalmology, 80336 Munich, Germany

Corresponding Author:

Anna Schuh, MD e-mail: anna.schuh@med.uni-muenchen.de

Supplementary Figure S1

Supplementary Figure S1 Annual number of enucleations for the most important causative diagnoses from 2013 to 2022.

Legend: The graph depicts the distribution of causative diagnoses for enucleations from 2013 to 2022. Causative diagnoses are represented on the y-axis, while the x-axis indicates the corresponding years. The trendline illustrates the stability of causative diagnoses during this period.

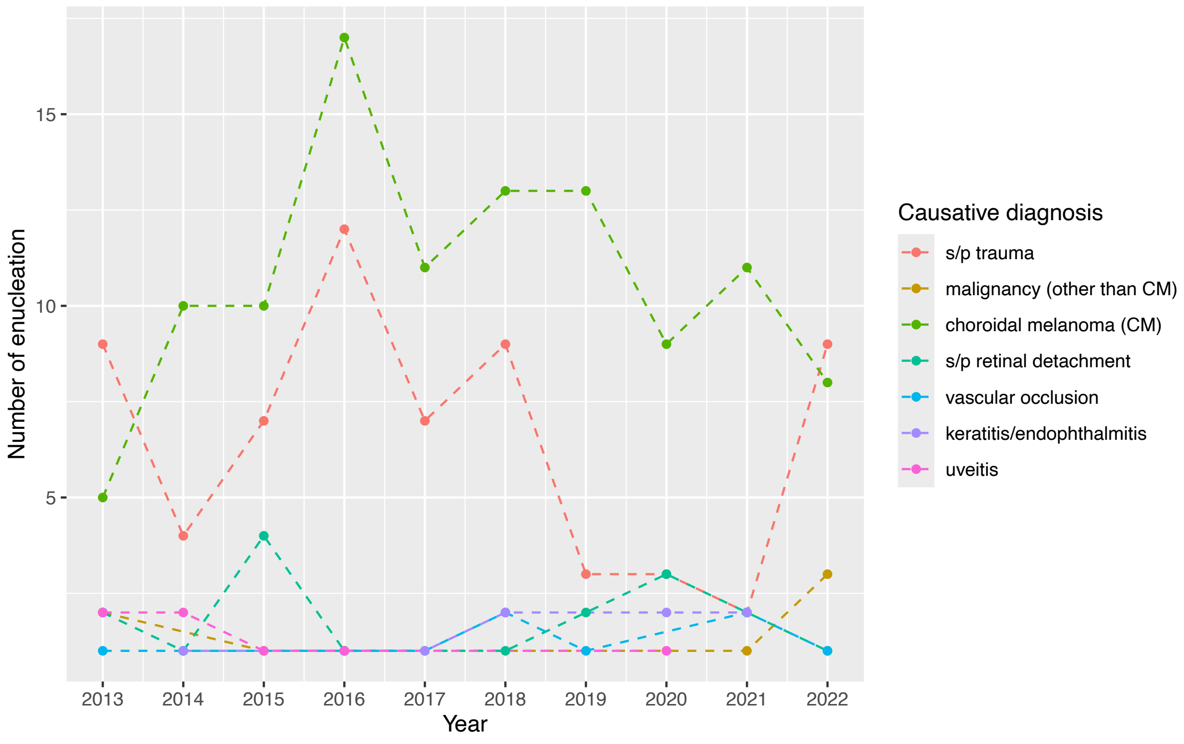

Supplementary Figure S2

Supplementary Figure S2 Differences of median age at enucleation regarding surgical diagnosis.

Legend: The graph illustrates significant differences in median age at enucleation based on surgical diagnosis. Surgical indications, such as malignancy, corneal perforation, and painful or disfigured blind eyes, are depicted on the x-axis, while the median age at enucleation is represented on the y-axis. Patients with malignancy and corneal perforation exhibit higher median ages compared to those with painful ($p < 0.001$ and $p = 0.002$, respectively, Wilcoxon rank-sum test) or disfigured blind eyes ($p = 0.002$ and $p = 0.013$, respectively).

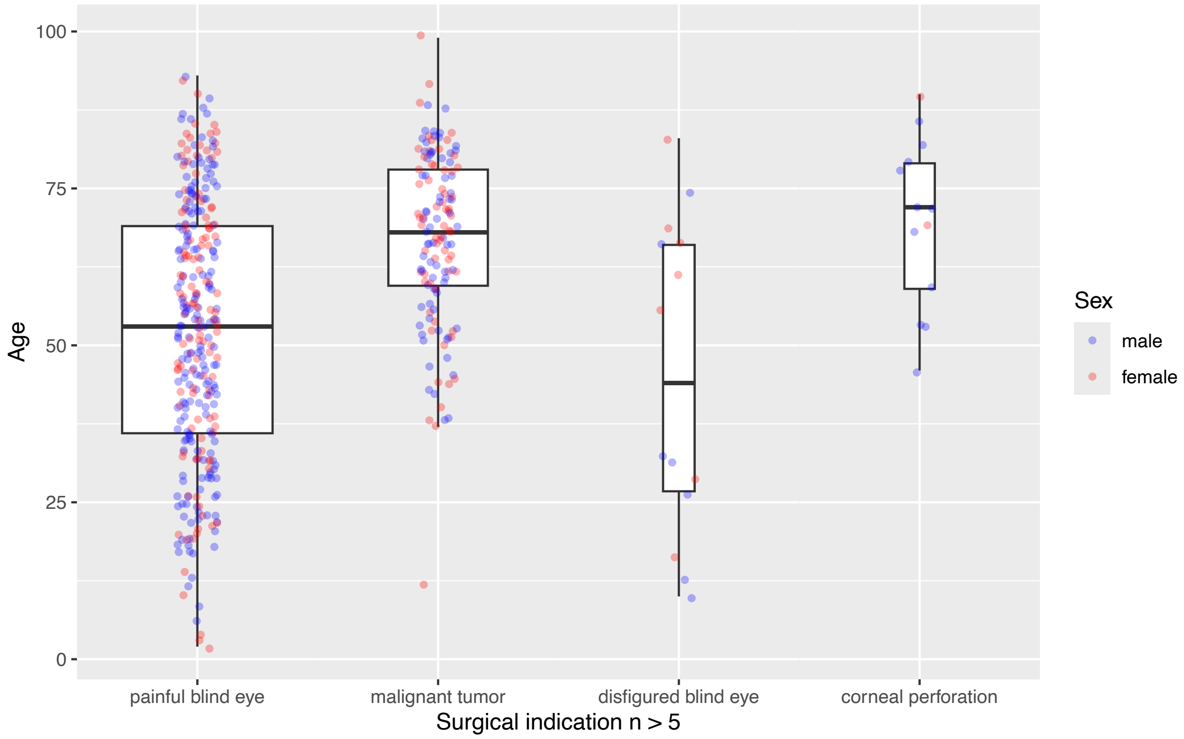

Supplementary Figure S3

Supplementary Figure S3 Differences of median age at enucleation regarding causative diagnosis

Legend: The graph reveals significant differences in median age at enucleation based on causative diagnosis. Causative diagnoses, including choroidal melanoma, previous trauma and retinal detachment, are displayed on the x-axis. The y-axis represents the median age at enucleation. Patients with underlying malignancy exhibit a higher median age compared to those with previous trauma or retinal detachment, with statistical significance (both $p < 0.001$, Wilcoxon rank-sum test).

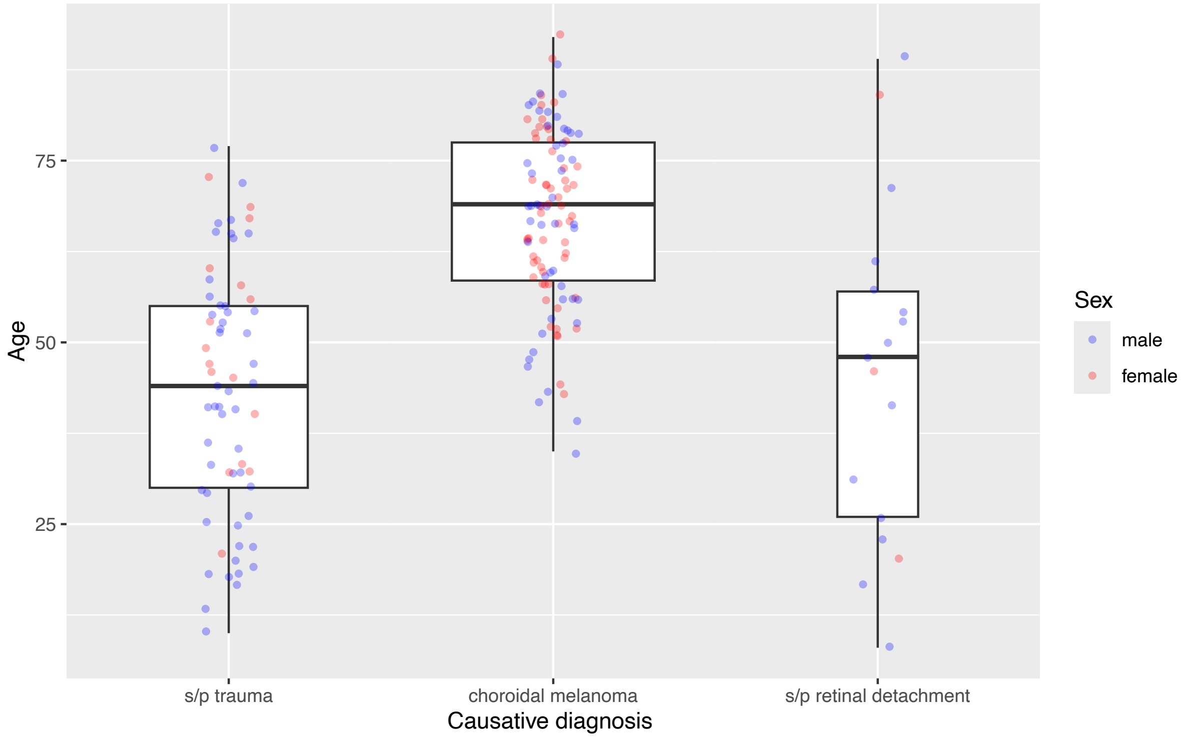

Supplement: Supplementary file 1 — Supplementary Information. [file 41598_2025_13975_MOESM1_ESM.pdf]
